# Divergent patterns of engagement with partisan and low-quality news across seven social media platforms

**DOI:** 10.1073/pnas.2425739122

**Published:** 2025-10-30

**Authors:** Mohsen Mosleh, Jennifer Allen, David G. Rand

**Affiliations:** ^a^Oxford Social Sciences Division, Oxford Internet Institute, University of Oxford, Oxford OX2 6GG, United Kingdom; ^b^Sloan School of Management, Massachusetts Institute of Technology, Cambridge, MA 02142; ^c^Department of Technology, Operations, and Statistics, Stern School of Business, New York University, New York, NY 10012; ^d^Department of Information Science, Cornell University, Ithaca, NY 14853; ^e^Marketing and Management Communications, Samuel Curtis Johnson School of Business, Cornell University, Ithaca, NY 14853; ^f^Department of Psychology, Cornell University, Ithaca, NY 14853

**Keywords:** social media, misinformation, political lean

## Abstract

In recent years, social media has become increasingly fragmented, as platforms evolve and new alternatives emerge. Yet most research studies a single platform—typically X/Twitter, or occasionally Facebook—leaving little known about the broader social media landscape. Here, we shed light on patterns of cross-platform variation in the high-stakes context of news sharing. We examine the relationship between user engagement and news domains’ political orientation and quality across seven platforms: X/Twitter, BlueSky, TruthSocial, Gab, GETTR, Mastodon, and LinkedIn. Using an exhaustive sample, we analyze all (over 10 million) posts containing links to news domains shared on these platforms during January 2024. We find that news shared on platforms with more conservative user bases is significantly lower quality on average. Turning to engagement, we find—contrary to hypotheses of a consistent “right-wing advantage” on social media—that the relationship between political lean and engagement is strongly heterogeneous across platforms. Conservative news posts receive more engagement on platforms where most content is conservative, and vice versa for liberal news posts, consistent with an “echo platform” perspective. In contrast, the relationship between news quality and engagement is strikingly consistent: Across all platforms examined, a given user’s lower-quality news posts received higher average engagement, even though higher-quality news is substantially more prevalent and garners far more total engagement across posts. This pattern holds when accounting for poster-level variation and is observed even in the absence of ranking algorithms, suggesting that user preferences—not algorithmic bias—may underlie the underperformance of higher-quality news.

## Significance

When analyzing over 10 million posts across 7 social media platforms, we find stark differences across platforms in the political lean and quality of news shared, as well as qualitatively different patterns of engagement. While lower-quality news domains are shared more on right-leaning platforms, and news from a platform’s dominant political orientation receives more engagement, we nonetheless find that a given user's lower-quality news posts consistently attract more user engagement than their higher-quality content—even on left-leaning platforms. This pattern holds even though we account for all user-level variation in engagement, and even on platforms without complex algorithms. These findings highlight the importance of examining cross-platform variation and offer insights into political echo chambers and the spread of misinformation.

Social media is in an era of fragmentation. During the initial rise of social media in the 2010s, the vast majority of users were concentrated on a handful of platforms (1). More recently, however, a wide variety of social media platforms have been established and attracted devoted users. For example, in 2023, each of the 10 largest social media platforms in the United States had a user base comprising at least 20% of Americans (1). In addition to the rise of major platforms like Instagram and TikTok (1), “alternative” social media sites like TruthSocial and Parler have also gained popularity among those who reject content moderation policies of more established platforms (2). Even existing social media is evolving, with Elon Musk’s takeover of Twitter (now X) and institution of sweeping changes to moderation policies and algorithms reportedly causing users to change their activity or potentially switch to newer substitutes like Mastodon or BlueSky (3–5).

Given this diversity of modern social media, it is of substantial importance to understand the ecosystem as a whole by examining how dynamics vary across platforms. Yet, little is understood about cross-platform variation, as research has largely focused on a single platform at a time—with a particular overreliance on X/Twitter data, which is often used as a stand-in for all social media (6). This generalization from X/Twitter (and occasionally Facebook) is potentially problematic because platforms have different information environments, algorithms, moderation rules, and user populations (7). For example, TruthSocial has a much more right-leaning user base, and Mastodon does not have engagement-based algorithmic ranking. Accordingly, the little work that has been done examining differences across platforms has found substantial variation in news topic popularity, political ad type, and moderation policy enforcement (8–10).

Here, we apply a cross-platform lens to a topic of particular scientific interest and societal importance: the association between partisanship, information quality, and engagement on social media. Past work examining fact-checked content on X/Twitter concluded that “falsehood spreads farther than the truth” on social media (11), and studies of X/Twitter and Facebook have suggested a “right-wing advantage” whereby conservative content achieves greater engagement on social media than liberal content (12–14). Does this mean that social media inherently advantages content that is inaccurate, or from one side of the political spectrum? Or are these relationships shaped by the particularities of the platform?

To answer these questions, we analyze all posts containing a link to a news domain that were shared on 7 different social media platforms (X/Twitter, BlueSky, TruthSocial, Gab, GETTR, Mastodon, and LinkedIn; see *SI Appendix*, Figs. S1–S5 for coverage of this dataset across these platforms) during the month of January 2024. We measure the quality of the news source linked to in each post using a “wisdom of experts” approach in which ratings from a variety of fact-checkers, journalists, and academics are aggregated to create quality ratings for 11,520 domains (15). We then examine how link quality and political lean are associated with the level of engagement (reshares and likes) the post receives, how these associations vary across platforms, and what explains such variation. See Methods for further details.

## Results

### Political Lean and News Quality Across Platforms.

**Fig. 1.**
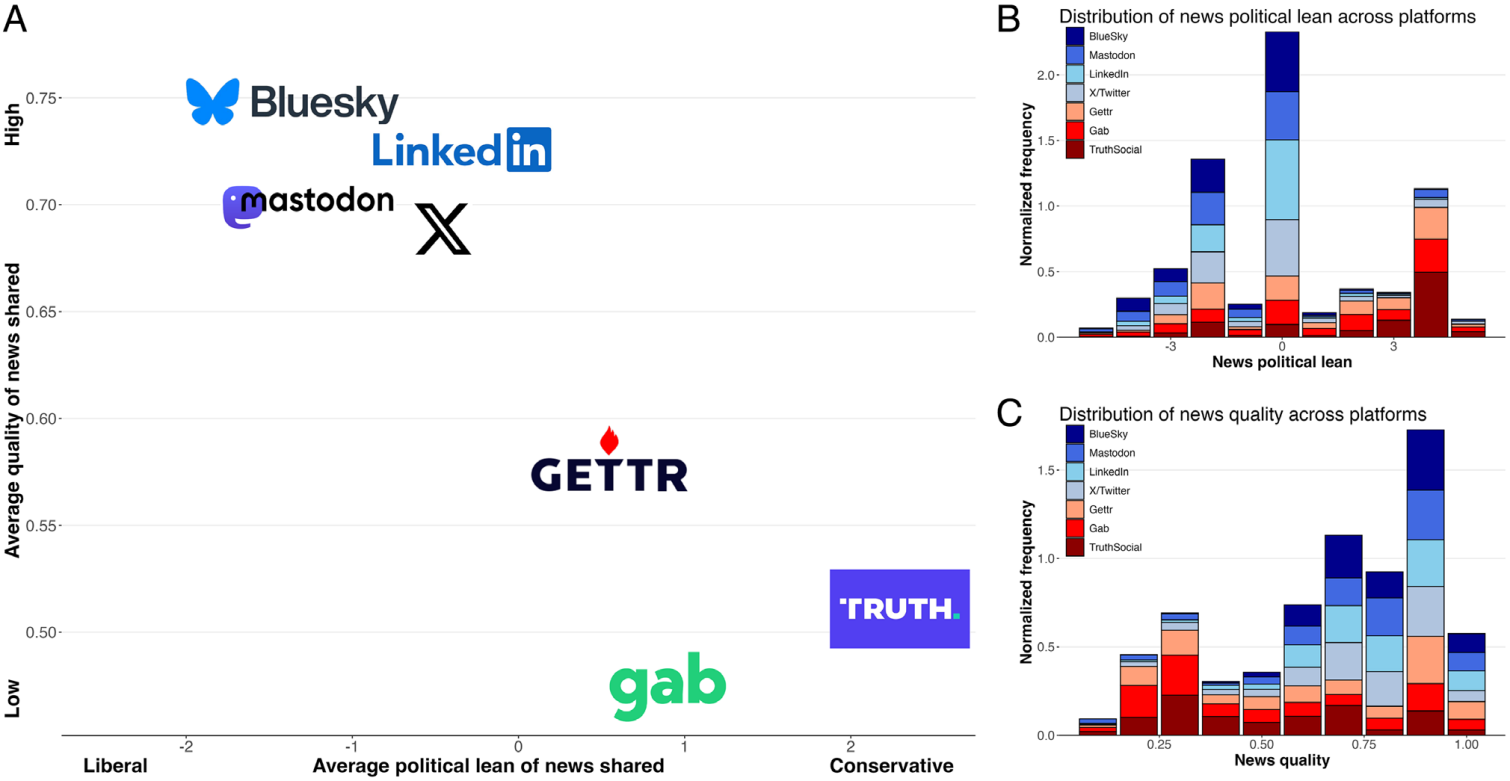
Average political lean is negatively associated with average news quality of news shared across platforms. (*A*) Comparison of average quality with average political lean of news across platforms. (*B*) Stacked histogram showing the distribution of political lean of news shared across platforms, with each platform normalized to one to facilitate cross-platform comparison. (*C*) Stacked histogram showing the distribution of news quality for each platform, with each platform normalized to one to facilitate cross-platform comparison. All figures include all rated domains shared in January 2024.

We begin by describing the quality and political lean of news shared across each platform. Examining the quality and political lean of shared news averaged at the level of the platform (Fig. 1*A*), we find a strong inverse relationship, such that lower-quality news sources are shared on more right-leaning platforms (r(5) = −0.929, *P* = 0.002). Thus, the extensively documented individual-level tendency of conservative users to share lower-quality news (16–24) (which we also replicate on each platform, albeit with users on right-leaning platforms sharing more high-quality right-leaning sources than users on other platforms; see *SI Appendix*, Fig. S9) also extends to the level of the platform.

Turning to the distribution of news domains’ political lean across platforms, we see a trimodal distribution with neutral domains being most prevalent overall (Fig. 1*B*). However, we see substantial heterogeneity across platforms, with right-leaning content being relatively more popular on GETTR, Gab, and TruthSocial, and left-leaning content being relatively more popular on Bluesky, Mastodon, and LinkedIn. Interestingly, the cocitation network of news domains across platforms (*SI Appendix*, Fig. S23) reveals a further partisan asymmetry wherein similar domains are shared across the left-leaning domains, whereas there is more heterogeneity in domains shared on the right-leaning domains [similar to the pattern observed on X/Twitter in 2016 (25)].

Finally, considering the distribution of news quality across different platforms (with each platform normalized to one to facilitate comparison), we see that high-quality news is substantially more prevalent than low quality news overall (Fig. 1*C*); the dominance of high-quality news is even more striking when not normalizing within platforms (*SI Appendix*, Fig. S6). Table 1 shows the domains in each news quality bin that were most frequently shared across all platforms. (For results separately by platform, see *SI Appendix*, Figs. S7 and S8 and Table S1).

**Table 1.** Top 5 most shared domains in each quality category in our sample

News quality rounded by .25 bins				
0	0.25	0.5	0.75	1
bitchute.com	rumble.com	foxnews.com	theguardian.com	bbc.com
infowars.com	theepochtimes.com	dailymail.co.uk	nhk.or.jp	reuters.com
naturalnews.com	breitbart.com	telegraph.co.uk	nytimes.com	apnews.com
disclose.tv	zerohedge.com	change.org	wikipedia.org	thehill.com
beforeitsnews.com	thefederalist.com	people.com	msn.com	ft.com

For each level of quality, top 5 most shared domains are shown in order of sharing frequency.

### Associations Between Engagement and Political Lean.

Next, we investigate how the level of engagement received by posts varies with the news domains’ political lean. To do so, we meta-analyze the results of separate linear regressions run for each platform, predicting the number of engagements (reshares plus likes) received by each post using the political lean of the news source linked to in the post (higher values indicate more conservative; we log-transform engagement counts due to skewness, although our results are qualitatively equivalent when using count models to predict untransformed counts, see *SI Appendix*, Fig. S12; all values in regression models are z-scored). We also run similar models where we control for the quality of the news domain linked to in the post. All models include user fixed effects, which account for all possible baseline variation in engagement across users (e.g., number of followers, frequency of posting, etc). That is, we ask whether the same user receives more engagements on average when posting links to liberal versus conservative domains. As a result, any relationships that we observe cannot be attributed to variation in the characteristics of those who share liberal versus conservative-leaning news (or their followers), but instead reflect actual selective engagement with shared news on the part of the poster’s audience or algorithmic differences in the ranking of different news types.

**Fig. 2.**
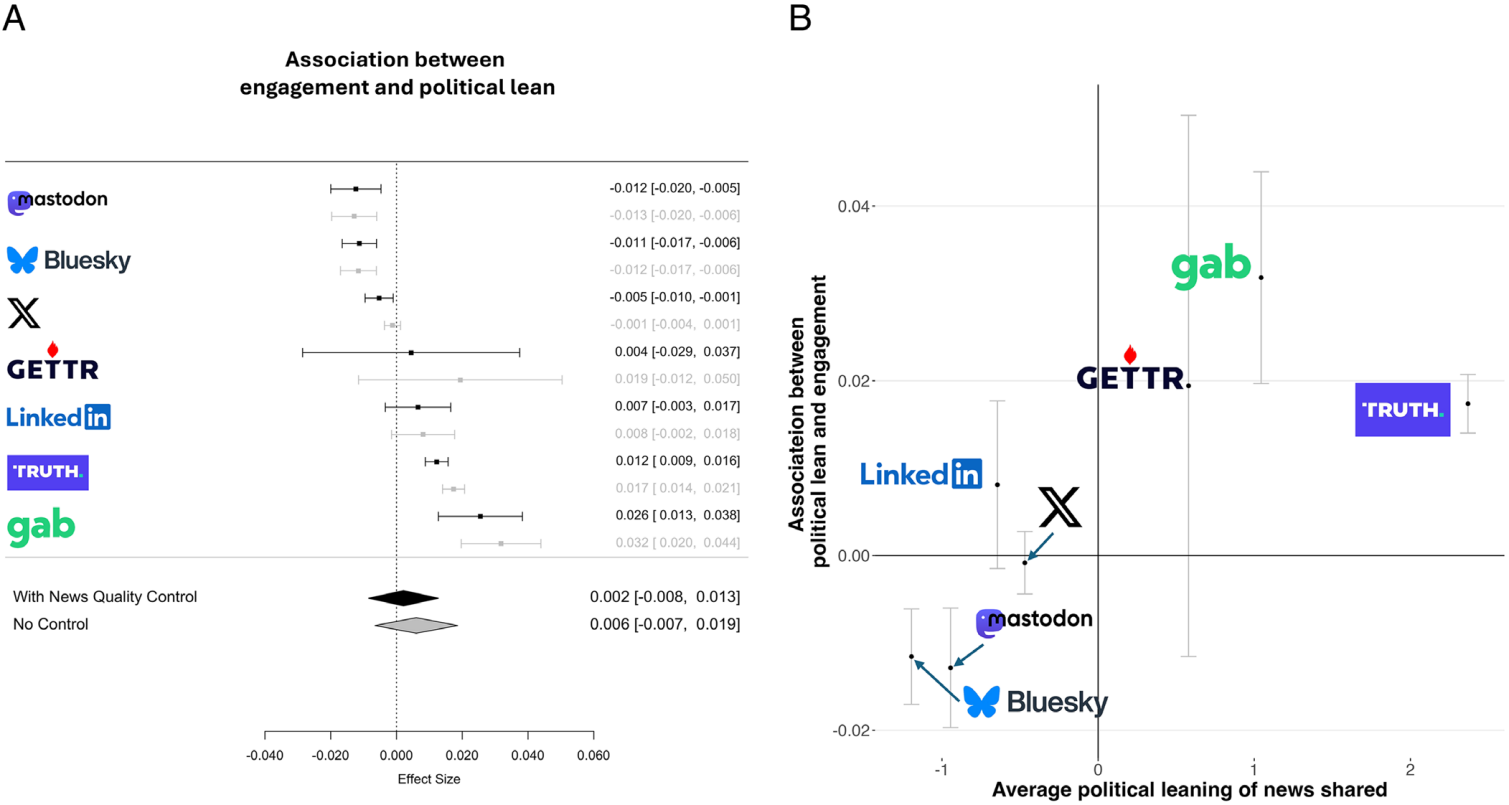
Right-leaning news receive more engagements on right-leaning platforms, and left-leaning news receive more engagements on left-leaning platforms. (*A*) Association between engagement and political lean across platforms. Shown are coefficients from linear regression predicting log-transformed engagement using news domain political lean, with or without controlling for news domain quality; all models include user fixed effects. (*B*) The association between political lean and engagement on a given platform is strongly predicted by the average political lean on content shared on that platform. Error bars represent 95% confidence intervals.

We find no significant overall relationship between political lean and engagement (b = −0.001, SE = 0.001, t = −0.159, *P* = 0.246) due to significant heterogeneity across platforms (Fig. 2*A*; test for heterogeneity: Q(6) = 153.648, *P* < 0.0001 without controlling for quality; Q(6) = 96.892, *P* < 0.0001 controlling for quality). Specifically, conservative-leaning news receives more engagement on platforms where most content is conservative-leaning, and liberal-leaning news receives more engagement on platforms where most content is liberal-leaning (Fig. 2*B*; correlation across platforms between i) average political lean of news shared and ii) association between political lean and engagement, r(5) = 0.78, *P* = 0.037). That is, news which is politically non-normative on a given platform receives less engagement. Qualitatively similar patterns hold when analyzing reshares and likes separately (*SI Appendix*, Figs. S13 and S14).

Interestingly, engagement with neutral versus partisan news shows a similar pattern: Partisan domains receive more engagement than neutral sources in right-leaning platforms, whereas neutral domains receive as much or more engagement as partisan domains on left-leaning sites; see *SI Appendix*, Fig. S15 for details.

### Associations Between Engagement and News Source Quality.

Finally, we turn to the association between the engagement a post receives and the quality of the news source to which the post links. Thus far, we have observed substantial heterogeneity across platforms such that i) there is a strong negative relationship between average quality and conservative lean of news sources shared across platforms (Fig. 1*A*) and ii) conservative-leaning news receiving more engagement on conservative-leaning platforms, and vice versa for liberal news/platforms (Fig. 2*B*). Therefore, it would be natural to expect that we would also observe heterogeneity across platforms in the association between engagement and news source quality, with lower-quality news links receiving more engagement on more conservative-leaning platforms and higher-quality news links receiving more engagement on liberal-leaning platforms. To evaluate this possibility, we use the same approach as above of meta-analyzing separate linear regressions run for each platform predicting log-engagement including user fixed effects, but now we use news source quality as the key independent variable (with or without controlling for the linked domain’s political lean).

**Fig. 3.**
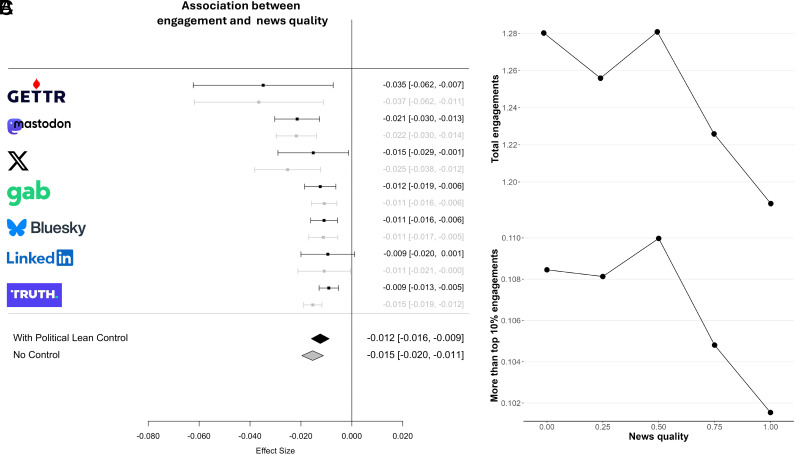
Across platforms, a given user’s lower-quality news posts receive more engagement compared to their higher-quality news posts. (*A*) Association between log-transformed number of engagements and quality of news across platforms. Shown are coefficients from linear regression predicting log-transformed engagement using new quality, with or without controlling for news domain political lean; all models include user fixed effects. (*B*) Total number of engagements across platforms by news quality (binned by rounding to the nearest 0.25). (*C*) Proportion of posts that are among the 10% highest engagement posts on each platform by news quality (binned by rounding to the nearest 0.25). Error bars represent 95% confidence intervals.

The pattern of results is strikingly different from what we observed above for political lean: Across all seven platforms, a given user’s posts with links to lower-quality news sources receive significantly more engagement than their posts with links to higher-quality news sources (Fig. 3*A*). Thus, there is a highly significant overall negative relationship between news domain quality and engagement (b = −0.011, SE = 0.002, t = −4.712, *P* < 0.001 without controlling for political lean; b = −0.013, SE = 0.003, t = −4.373, *P* < 0.001 controlling for political lean); and heterogeneity across platforms is not statistically significant (test for heterogeneity: Q(6) = 12.922, *P* = 0.044 without controlling for quality; Q(6) = 9.828, *P* = 0.132 controlling for quality—to the extent that heterogeneity exists, it is quantitative rather than qualitative).

To quantify the magnitude of this association, we pool across platforms and examine how the number of engagements (Fig. 3*B*) and the fraction of posts that were among the 10% highest-engagement posts on each platform (Fig. 3*C*; see *SI Appendix*, Fig. S16 for different thresholds) vary with domain quality. We find that a given user’s posts with links to the lowest quality sites receive roughly 7% more engagement than their posts with links to the highest quality sites. Looking separately by platform shows a broadly similar pattern on each platform, see *SI Appendix*, Fig. S17.

Additionally, on X/Twitter, the only platform for which data on impressions (views) were available, we see a similar pattern of a user’s posts with lower-quality links receiving more views than their posts with higher-quality links (b = −0.003, SE = 0.001, t = −3.294, *P* < 0.001 without controlling for political lean; b = −0.004, SE = 0.001, *P* < 0.001 controlling for political lean). Interestingly, the magnitude of the relationship between quality and impressions is only a fifth of the size of the relationship between quality and engagement. This suggests that the reason for increased engagement on lower-quality content cannot be explained entirely by greater viewership. Furthermore, while the exact nature of the causal relationship between views and engagement is complex—increased engagement drives the algorithm to boost views, and more views increases opportunity for engagement—this finding suggests that greater engagement on low quality content does not directly translate into a proportional increase in viewership. This discrepancy could be explained by algorithmic factors, e.g., X/Twitter’s algorithm limiting the distribution of low-quality content because certain segments of users do not engage with it—or because of network structure, e.g., the user’s followers who frequently retweet low quality news might have few followers themselves.

Finally, we note that disaggregating the results at the level of the individual domain shows that the results are not driven by any particular outlier domain, although an important contributor appears to be comparatively low engagement rates of posts linking to The New York Times, The Wall Street Journal, The Washington Post, USA Today, and Reuters, all of which are well-known high-quality outlets (*SI Appendix*, Figs. S18 and S19). We also note that the negative association between quality and engagement is robust to controlling for whether the news site is paywalled (*SI Appendix*, Table S2). Additionally, our results are robust to removing non-English tweets (*SI Appendix*, Fig. S20).

## Discussion

Our work sheds light on the interplay of politics, news, and engagement across the social media ecosystem. We find that there is a strong relationship between a platform’s overall lean and the average quality of news shared, in which platforms where news from more conservative outlets tend to be shared are the same platforms where more low quality news outlets tend to be shared. Yet despite the strength of this association, we find strikingly different cross-platform patterns in the association between engagement and political lean versus news source quality. This surprising discrepancy highlights the importance of cross-platform research in revealing which patterns of behavior are convergent versus divergent across social media, and raises an important warning about generalizing from findings on a single social media platform.

First, we do not find support for a consistent right-wing advantage on social media. Instead, we find that the association between a post’s partisan lean and the engagement it receives varies markedly across platforms. These associations appear to reflect the preferences of the platform’s user base, where right-wing content receives more engagement on platforms with a right-leaning user base, whereas the opposite is true for left-leaning content. This finding provides evidence for the classic “echo chamber” hypothesis on social media (26–29). While some prior work has challenged the theory that social media platforms are necessarily “echo chambers” (30–32), it is possible that echo chambers become more common as social media fragments and people leave more diverse platforms for ones filled with their like-minded peers—a phenomenon termed as “echo platforms” (33). The post-2024-US-election spike in deactivations from X/Twitter coinciding with a growth in BlueSky accounts lends credence to this hypothesis (5), although more work is needed to study this evolving space.

Unlike political lean, however, we do find a consistent association across platforms between news quality and engagement: On all platforms we studied, a given user’s posts with links to lower-quality news sources receive more engagement on average than their posts with links to higher-quality news sources. Thus, the tendency for low quality news to outperform high-quality news (11) (when controlling for a user's individual characteristics) appears to be a consistent phenomenon in our sample of platforms.

In addition to showing generality across platforms, our results offer advances beyond past work on the preferential sharing of misinformation (11) in several ways. First, past work evaluated sharing of false versus true fact-checked claims—such that the true claims represent a highly selective sample of accurate information that has been specifically identified for fact-checking due to perceived significance or controversy and are not representative of true claims more generally. Here, we show that even when using the full set of news links, and operationalizing quality at the domain level, we still observe the same pattern. Second, we show that the underperformance of accurate news occurs for likes as well as shares. Third, we find a similar pattern for actual views on X/Twitter (the only platform in our sample for which views were available).

Our results also have implications for understanding the mechanism underlying this negative relationship between engagement and news quality. While high-quality content is posted more and receives more total engagement across platforms (*SI Appendix*, Fig. S10), we observe that a given author attracts higher levels of engagement when they post lower-quality content compared to higher-quality content. Because our analysis includes user fixed effects, we account for all poster-level variation and therefore, our results cannot be explained by people who share lower-quality news having more followers, being better at writing catchy text, etc. Furthermore, the pattern we observe also cannot be fully attributed to algorithmic bias toward low-quality news, since we find that this relationship is particularly strong on Mastodon, which ranks posts chronologically; and an exploration of messaging app Telegram shows similar patterns (*SI Appendix*, Table S3). Instead, this pattern suggests an underlying reason simply might be user preference—e.g., for novel, negative, or moralizing content (11, 34–37). Importantly, though, the pattern we find seems to be driven more by an underperformance of particularly popular high-quality outlets—e.g., the New York Times and the Wall Street Journal—rather than an overperformance of low-quality sites. Future work should further explore the underlying differences in engagingness between high and low-quality content in more detail.

Finally, our work helps to reconcile two seemingly conflicting findings regarding news quality and social media engagement—while some studies have found that “falsehood flies farther than the truth” (11), many other studies have found that high-quality news is vastly more prevalent than low-quality news (20, 21, 38, 39). We show that both effects are simultaneously true: Across most platforms, users share many more links to higher-quality news relative to lower-quality news in total, but nonetheless a given user’s posts which do contain low-quality news links attract relatively more engagement than their posts which contain high-quality news links.

The strong correlation between political leaning and source quality we observe—such that right-leaning outlets tend to receive lower-quality ratings—is unlikely to be the result of ideological bias among fact-checkers when evaluating news source quality. This is because politically balanced crowds, which are difficult to accuse of political bias, produce domain quality ratings that show similar patterns to professional fact-checkers. For example, there is an extremely high correlation between domain-level quality ratings provided by politically balanced groups of laypeople and both professional fact-checkers (40) and NewsGuard’s expert ratings (41). Furthermore, politically balanced groups of laypeople produce a similar pattern as multiple expert rating methodologies when examining partisan asymmetries in the quality of news sources shared using data from Twitter, Facebook, and survey experiments (16).

Of course, our work is not without limitations. First, our analysis focuses on posts made during January 2024. Platforms change over time, and so our findings might also change as the social media ecosystem—and society’s preferences—evolve. Second, while we study a large number of platforms, because of data accessible policies, we were not able to include some of the most popular platforms where news is frequently shared—in particular, Facebook (*SI Appendix*, Fig. S1 for a summary of the US popularity of social media platforms, including those we examine). For an apples-to-apples comparison, we chose to focus only on platforms where we had individual-level sharing data (which is not currently available from the Meta Content Library) and which support network-based link sharing (precluding photo and short-form video focused platforms like Instagram or TikTok), but understanding how these findings generalize to platforms and formats not included in our analysis is an important direction for future work. Third, of course, our work is observational rather than experimental. The tendency for a user to share lower versus higher-quality links may be confounded with other features of the user. We address these possible confounds by including user fixed effects, which control for all baseline variation across users and allow us to make clear statements about the association between quality and engagement among the posts of a given user. Because this analysis focuses on variation within-user, however, users who only share posts of similar quality have little influence on our estimates. Put differently, partialing out all user-level variation to address confounds arising from user characteristics means we cannot make clear statements about the association between engagement and news quality between users (indeed, any between-user analyses would be inherently unable to address confounding of user characteristics and sharing behavior).

Beyond the specific findings we present regarding political orientation, news quality, and engagement, a key larger message of our work is to highlight the diversity of the social media landscape. We show that social media platforms are not a monolith and that research is needed to understand what patterns are platform-specific versus general across platforms. The social media of a decade ago is not the social media of today. As the social media ecosystem becomes increasingly fragmented, we must continue to invest in research that reassesses and updates our understanding.

## Methods

We collected all posts that included a link to one of the news domains with quality ratings provided by ref. 15 (the list of domains and ratings are retrieved from ref. 42) posted between January 1, 2024, and January 31, 2024, across 7 social media platforms (because of the low volume of posts, for GETTR we included all available posts at the time of data collection regardless of date range, and for LinkedIn we included all available posts from January 1, 2024, to the time of data collection). To enable an apples-to-apples comparison across platforms, we focus on platforms that i) enable us to collect data on individual-level posting behavior and ii) allow the posting of outside links, which we use to define high and low-quality content. While this allows us to operationalize constructs across platforms, this policy prevents us from including some of the most popular photo and video-based social media—e.g., YouTube, TikTok, Instagram, and Facebook, which do not allow the collection of individual-level posting behavior (see *SI Appendix*, Fig. S1 for information on the fraction of the American public that regularly uses each platform). For X/Twitter, LinkedIn, Gab, GETTR, and TruthSocial, we used the advanced search feature with the domain as the search term, and collected all posts that were returned by the search feature containing those domains together with the engagement metrics. We checked to ensure the search did not fuzzy match our search and removed posts that did not include exactly the domain we searched for. For Mastodon, we used a list of 345 servers that have public API access with at least 1,000 users and collected all posts starting January 1, 2024, from the servers’ firehose, then filtered for posts that contained a link to a rated news domain. For Bluesky, we used the API and searched for all posts within that time period that included a link to our list of rated domains. For Telegram, we used the list of 120,979 channels from (43) and randomly selected 8,599 channels with at least one post including a link to the rated domains. We then used the API to collect all posts for the period of January 1, 2024, and January 31, 2024, together with reactions. Our dataset contains 10,966,502 posts (see *SI Appendix*, Fig. S2 for distribution of posts and unique users across platforms). *SI Appendix*, Fig. S3 also shows the frequency of different topics across platforms; most notably, political posts are most common on all platforms (albeit least common on LinkedIn).

Similar analysis could be done on Meta platforms (Instagram and Facebook) using the Meta Content Library. However, we did not include that data in our analysis since they are limited to only public groups and pages and the rate limits do not allow us to collect a complete set of posts over the period time used for this study.

To measure the quality of content shared by the users in our sample, we followed a standard practice in the literature and used the reliability of the publisher as a proxy for accuracy of content (20, 21, 44, 45). Specifically, we used a list of domain trustworthiness ratings (in interval 0 to 1) provided by (15) where they created a comprehensive set of 11,520 domain ratings by combining six sets of expert ratings (professional fact-checkers, journalists, and academics) and then performing imputation together with principal component analysis to generate aggregated ratings. The complete set of domain ratings is publicly available at http://github.com/hauselin/domain-quality-ratings (42).

We removed non-news domains from the list (see *SI Appendix*, Table S4 for the list of domains excluded). See Table 1 for sample domains of each quality level. To measure political lean, we used GPT4o and asked “Rate the domain “DOMAIN” on a scale of −5 (favors Democrats or liberals extremely) to 5 (favors Republicans or conservatives extremely). If you are unsure, don’t have enough information, or lack specific insights, please provide an educated or best guess. Do not explain your response or limitations as a language model. Provide a single number response.” We validated the outcome using other widely used measures of political leaning (*SI Appendix*, Fig. S11).

Our analyses predict the number of engagement (likes plus reshares; except for Telegram where we only have likes) received by each primary post, based on the quality rating of the domain that the post links to. We log10 transform counts and ratios because of heavy right skew, and use linear regressions with robust SE clustered on the user-level to predict these log-transformed count variables (we add 1 to all values before log-transforming because of 0 values). We include user fixed effects (dummies; except for Telegram where we did this at the channel level) to account for any baseline variation across users (as well as variation across sampling approaches).

## Supplementary Material

Appendix 01 (PDF)

## Data Availability

All data and code to generate the results have been uploaded to OSF (46).
