# Supplementary material for: Divergent patterns of engagement with partisan and low-quality news across seven social media platforms
Source: Proc Natl Acad Sci U S A. 2025 Oct 30;122(44):e2425739122. doi: 10.1073/pnas.2425739122 (PMC12595477; doi:10.1073/pnas.2425739122)
Supplement: Supplementary file 1 — Appendix 01 (PDF) [file pnas.2425739122.sapp.pdf]

Supplementary Information
for
Divergent patterns of engagement with partisan and low-quality news across seven social media platforms

Mohsen Mosleh[1,2]*, Jennifer Allen[3], and David G. Rand[2,4,5,6]

[1]Oxford Internet Institute, University of Oxford, United Kingdom; [2]Sloan School of Management, Massachusetts Institute of Technology, United States; [3]Department of Technology, Operations, and Statistics, NYU Stern School of Business, New York, United States, [4]Department of Information Science, Cornell University, United States; [5]Marketing and Management Communications, SC Johnson School of Business, Cornell University, Ithaca, United States; [6]Department of Psychology, Cornell University, Ithaca, United States

*Corresponding author: mohsen.mosleh@oii.ox.ac.uk

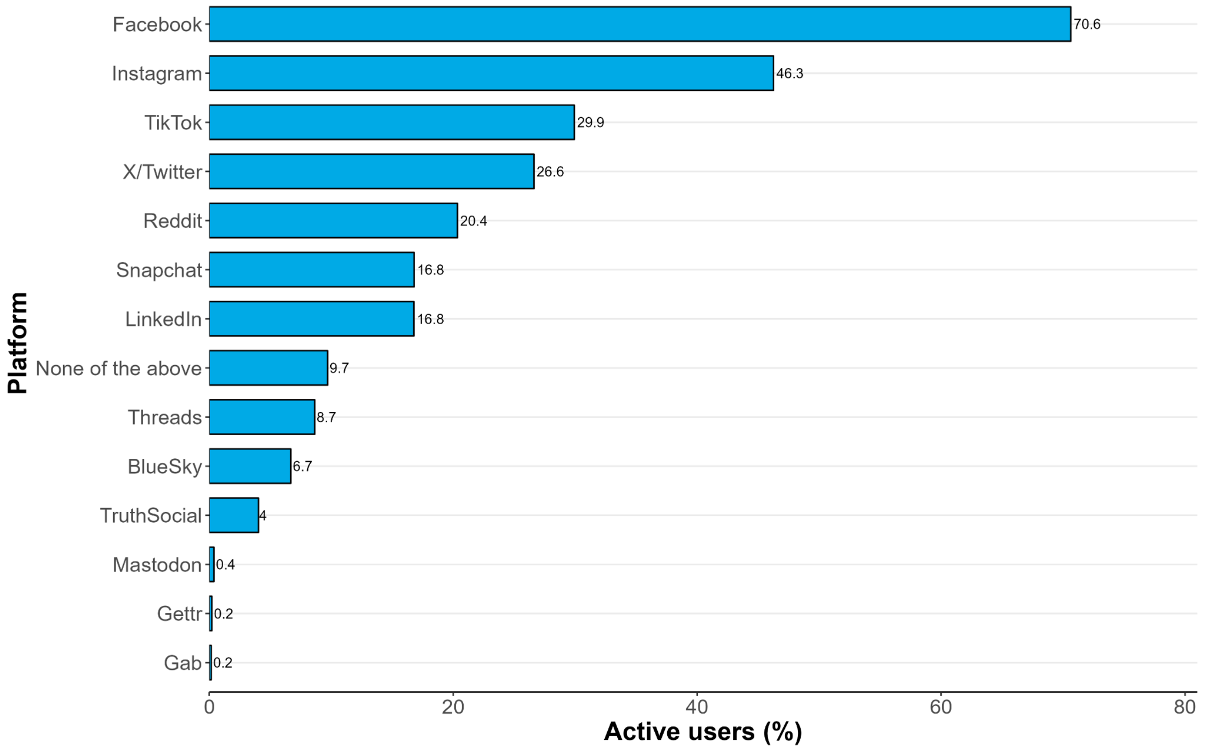

Figure S1. Estimated use of different social media platforms using national representative samples. *We conducted a survey on YouGov where we interviewed 2,131 respondents who were then matched down to a sample of 2,000 to produce the final dataset. The prompt was "Which, if any, of the following platforms do you regularly use?" The respondents were matched to a sampling frame on gender, age, race, and education. The sampling frame is a politically representative "modeled frame" of US adults, based upon the American Community Survey (ACS) public use microdata file, public voter file records, the 2020 Current Population Survey (CPS) Voting and Registration supplements, the 2020 National Election Pool (NEP) exit poll, and the 2020 CES surveys, including demographics and 2020 presidential vote. The matched cases were weighted to the sampling frame using propensity scores. The matched cases and the frame were combined and a logistic regression was estimated for inclusion in the frame. The propensity score function included age, gender, race/ethnicity, years of education, and region. The propensity scores were grouped into deciles of the estimated propensity score in the frame and post-stratified according to these deciles. The weights were then post-stratified on 2020 and 2024 presidential vote choice as well as a four-way stratification of gender, age (4-categories), race (4-categories), and education (4-categories), as well as a two-way stratification on race (4-categories) and education (4-categories) to produce the final weight.*

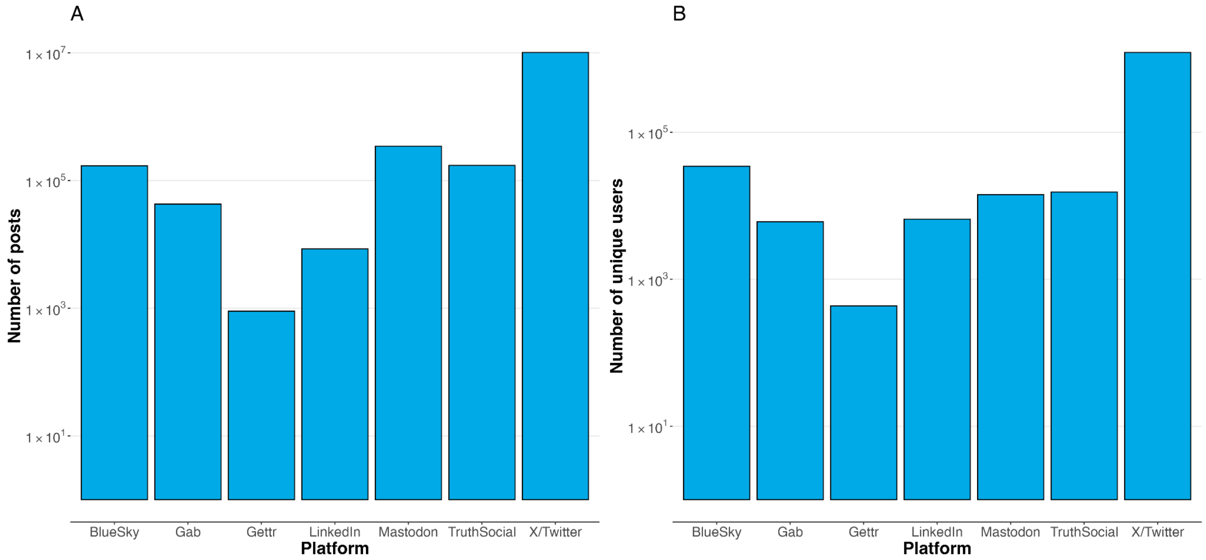

Figure S2. (A) Number of posts, and (B) number of unique users for each platform in our sample.

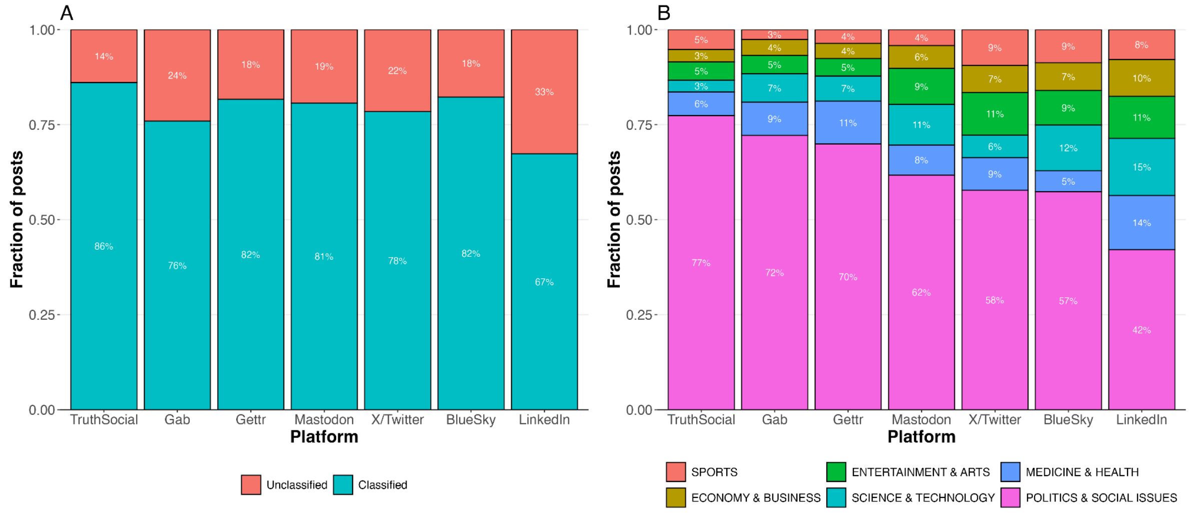

Figure S3. Prevalence of different topics across platforms. *We extracted the news headline of all URLs shared across the platforms (except for X/Twitter and Mastodon where we quota sampled based on news quality due to the large number of URLs) using online service Diffbot.com. We then used GPT-4o-mini to classify headlines into 6 topics. (A) Fractions of posts for which the news headlines were classified. (B) Fraction of posts containing each topic across all platforms for classified posts. Platforms are sorted based on % of posts including headlines related to politics.*

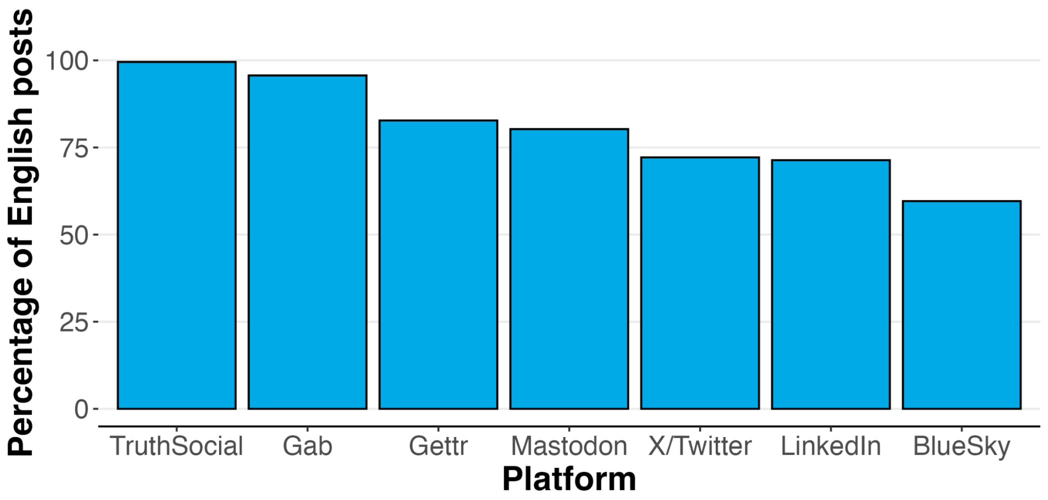

Figure S4. Percentage of English posts across platforms. *We used the Python language detection package (https://pypi.org/project/langdetect/) to find whether the post was written in the English language.*

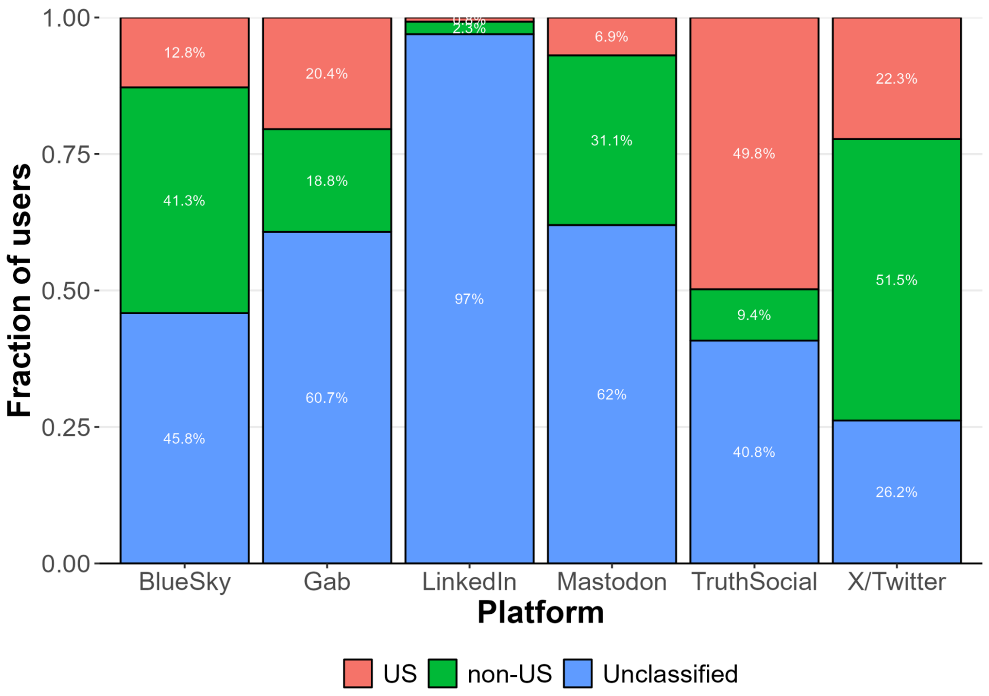

Figure S5. Location of users identified from their profile. *We used GPT-4.1-nano to identify users' location using their bio description and location (if available) self-reported on the platform (GETTR not included since we could not obtain relevant information for the users).*

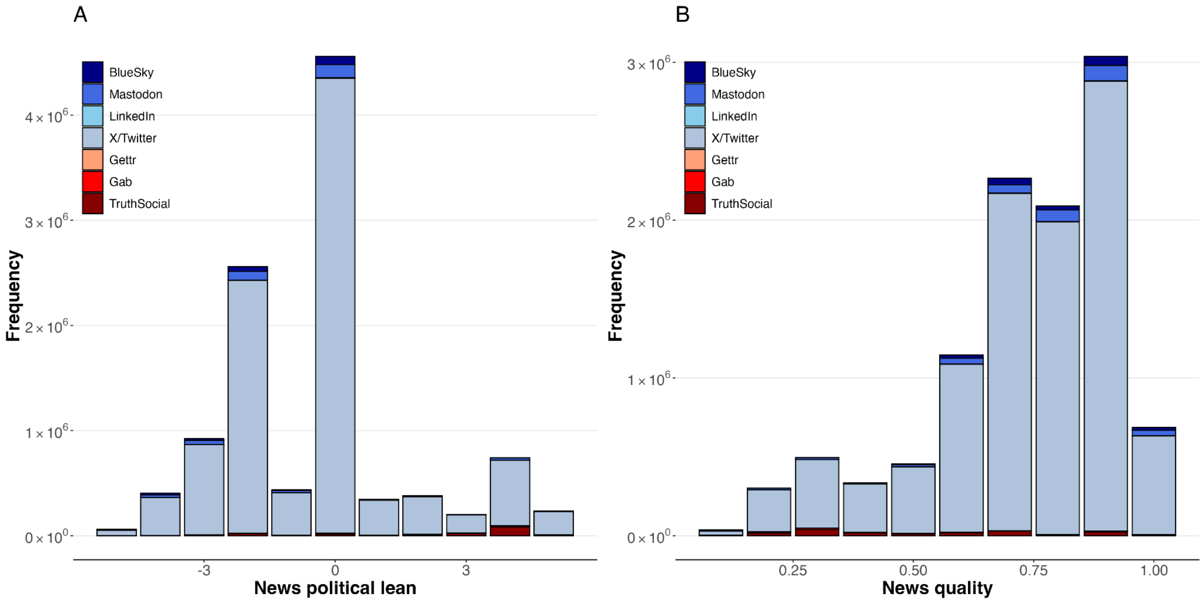

Figure S6. Frequency distribution of (A) news quality and (B) political lean of news domains shared across platforms, without normalizing within each platform.

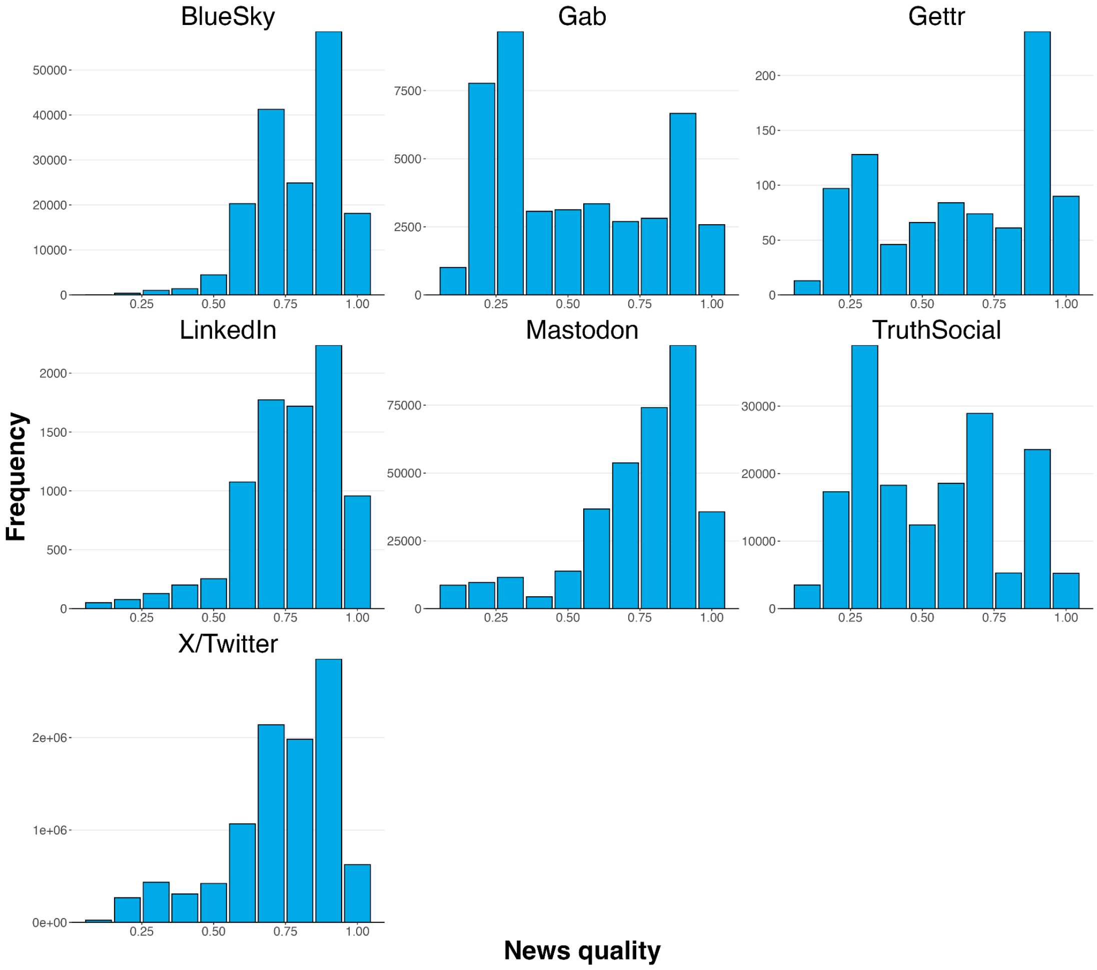

Figure S7. Frequency distribution of quality of news shared on each platform.

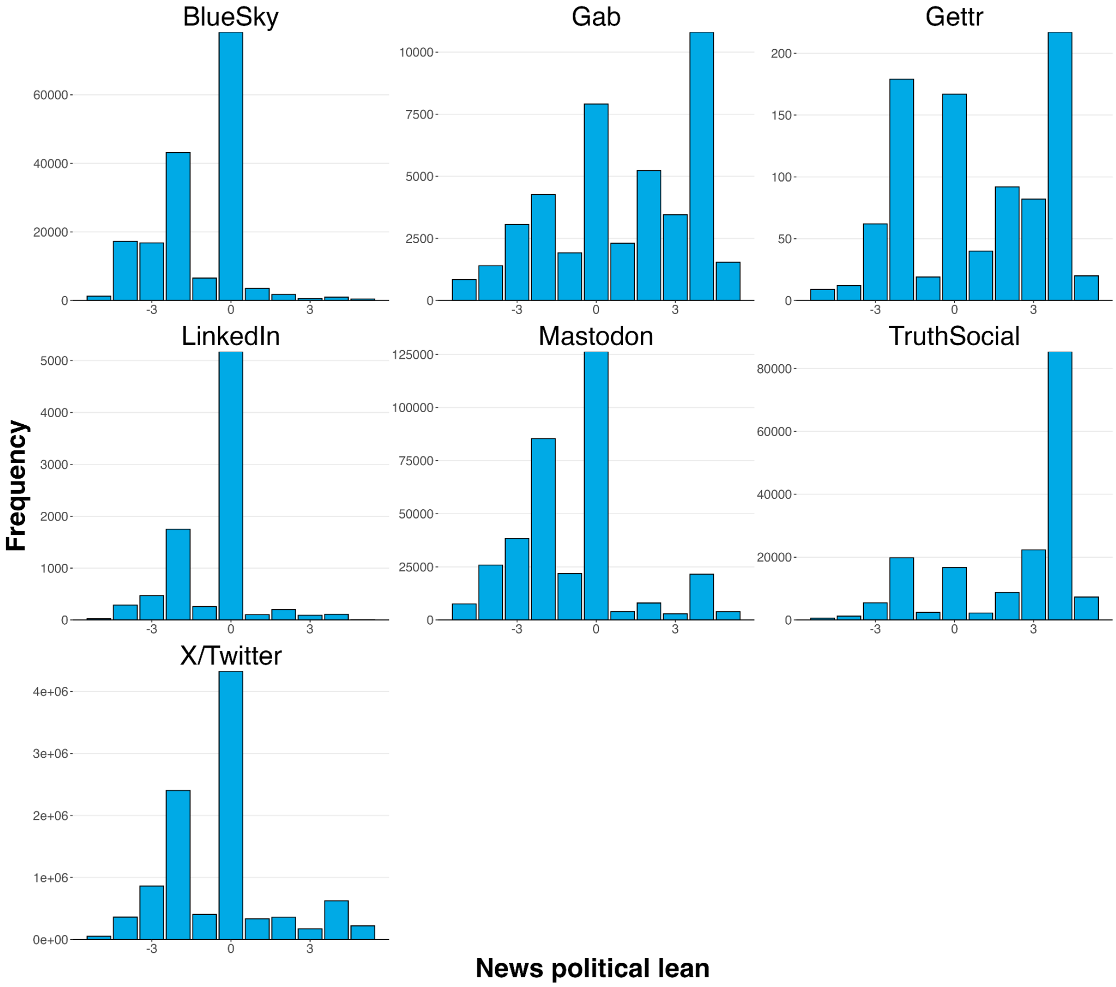

Figure S8. Frequency distribution of political lean of news domains shared on each platform.

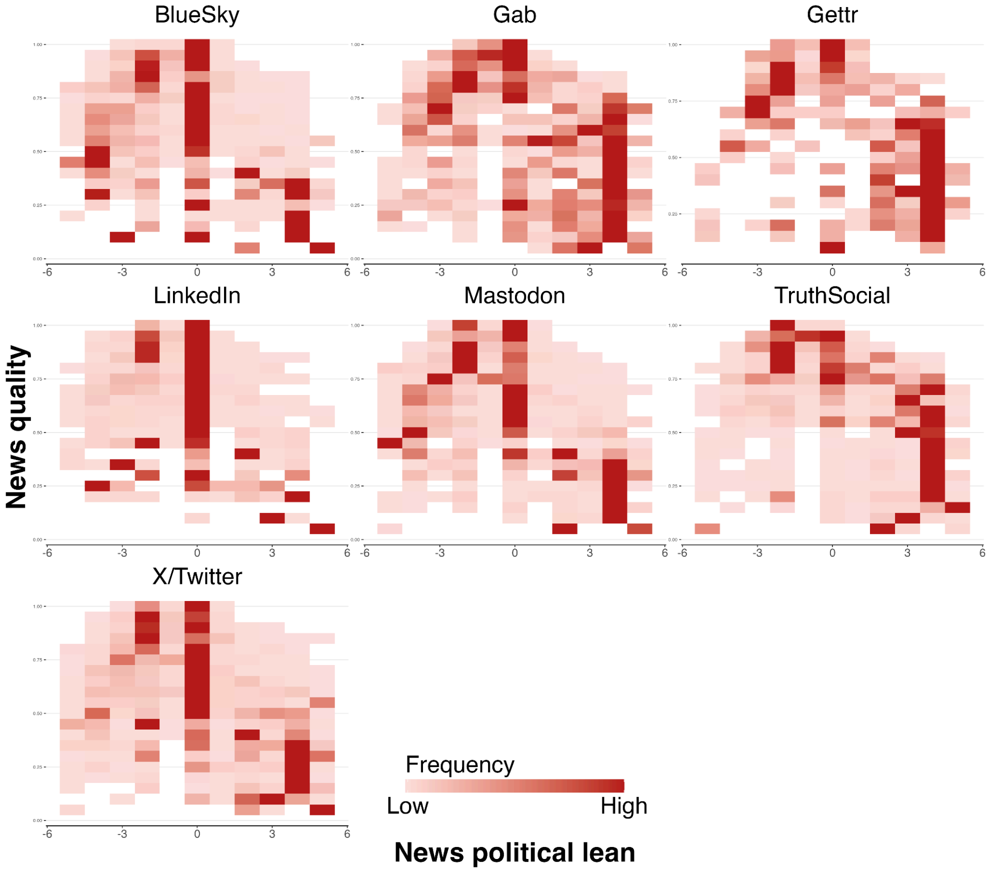

Figure S9. Joint distribution of news quality and political lean. *Intensity of colors shows frequency of news domains shared per each cell (darker color = higher frequency sharing). Results reveal an interesting contrast between platforms—on the right-leaning platforms (Gab, GETTR, and Truth Social), users share similar amounts of lower-quality and higher-quality right-leaning news, whereas on the other platforms most right-leaning content that is shared is lower-quality (i.e., the higher-quality right-leaning domains are rare on the other platforms). As a result, right-leaning content appearing on left-leaning platforms tends to be of lower quality than right-leaning content appearing on right-leaning platforms.*

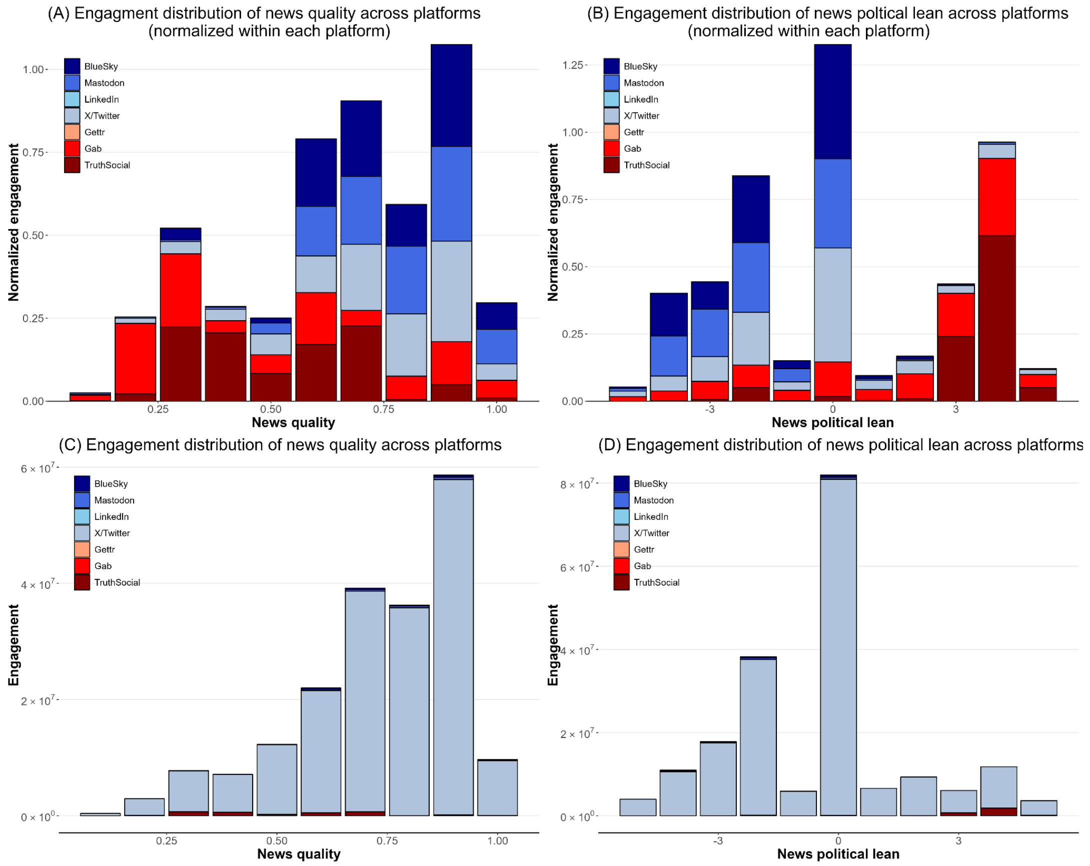

Figure S10. Engagement distribution of news quality and political lean. *(A) Stacked histogram showing the engagement distribution of news quality across platforms, with each platform normalized to one to facilitate cross-platform comparison. (B) Stacked histogram showing the engagement distribution of news political lean across platforms, with each platform normalized to one to facilitate cross-platform comparison. (C) Stacked histogram showing the engagement distribution of news quality across platforms. (D) Stacked histogram showing the engagement distribution of news political lean across platforms.*

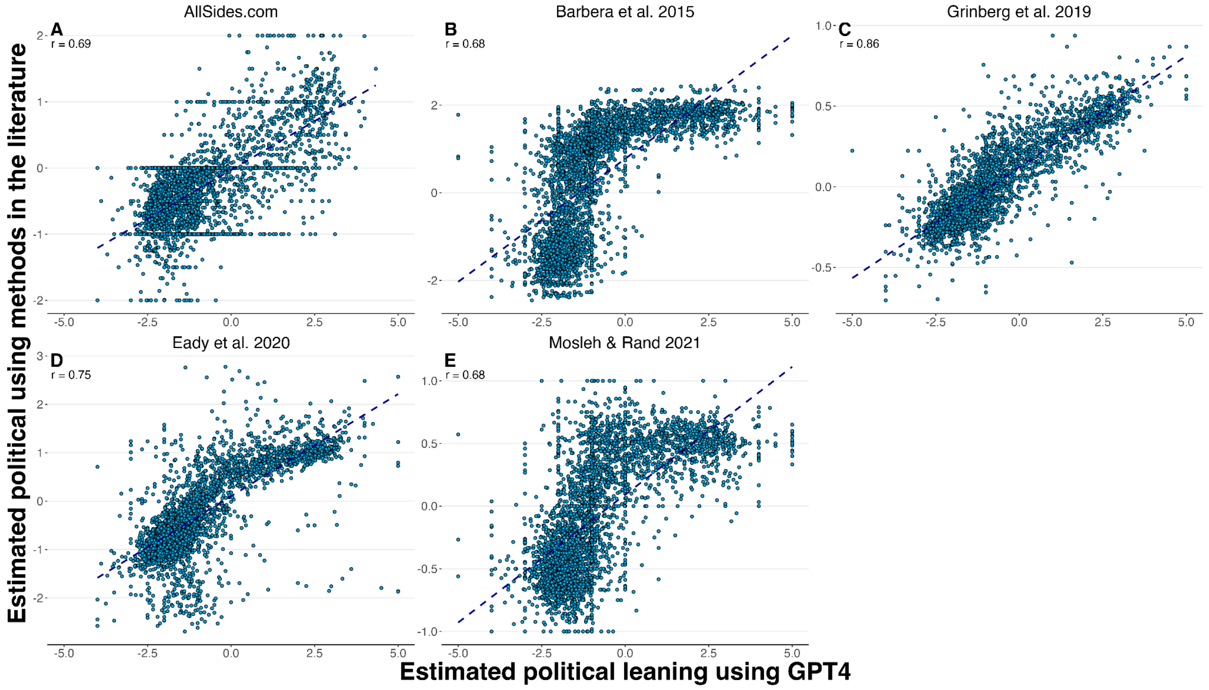

Figure S11. Correlation of estimated political lean using GPT4o and other methods for estimating political lean in the literature. *Results are generated using a dataset of 5,000 X/Twitter users from (1). Shown here is correlation of users' political lean estimated by the average political lean based on the news domain they shared, and (A) users' political lean estimated by averaging the lean of the domains provided by allsides.com, (B) users' political lean estimated using the statistical method provided by (2) based on the accounts users follow, (C) users' political lean estimated by averaging the lean of the domains provided by (3), (D) users' political lean estimated using the statistical method from (4) based on the news domain they shared, (E) users' political lean estimated using the method from (1) based on political elites the users follow.*

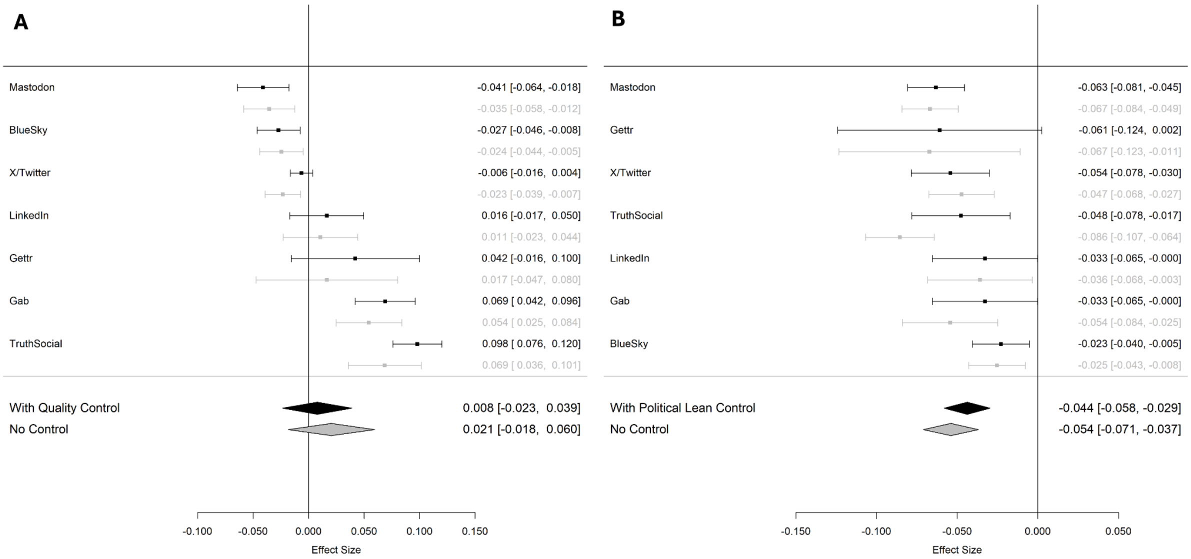

Figure S12. Robustness of results using a count model. *Negative binomial regression predicting engagement count using (A) news domain political lean, and (B) news domain quality, with or without controlling for news domain quality/political lean; all models include user fixed effects.*

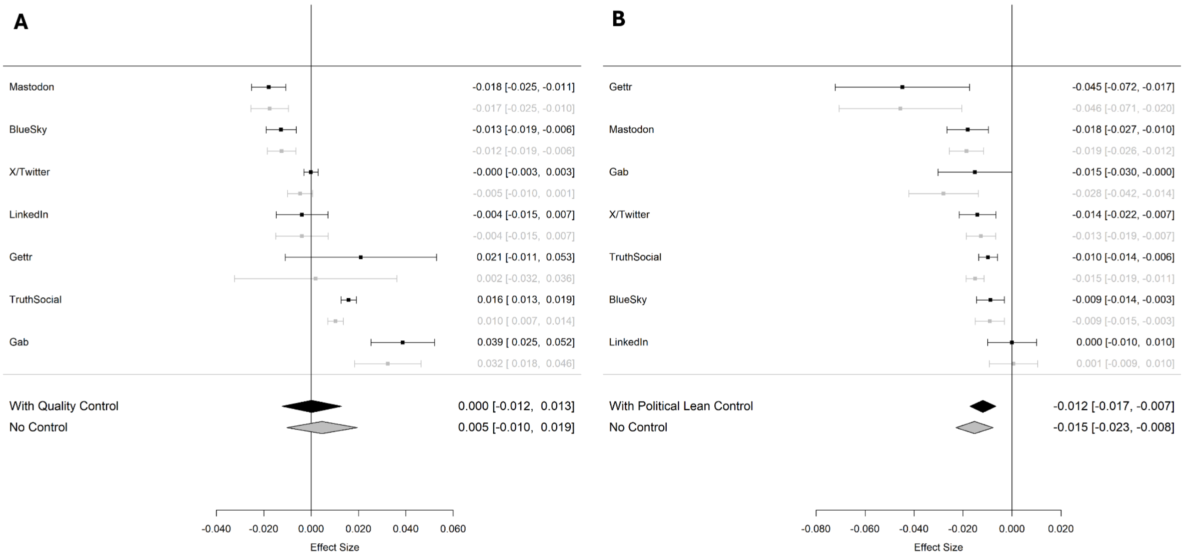

Figure S13. Robustness of results predicting re-shares only. *Linear regression predicting log-transformed number of reshares of (A) news domain political lean, and (B) news domain quality, with or without controlling for news domain quality/political lean; all models include user fixed effects.*

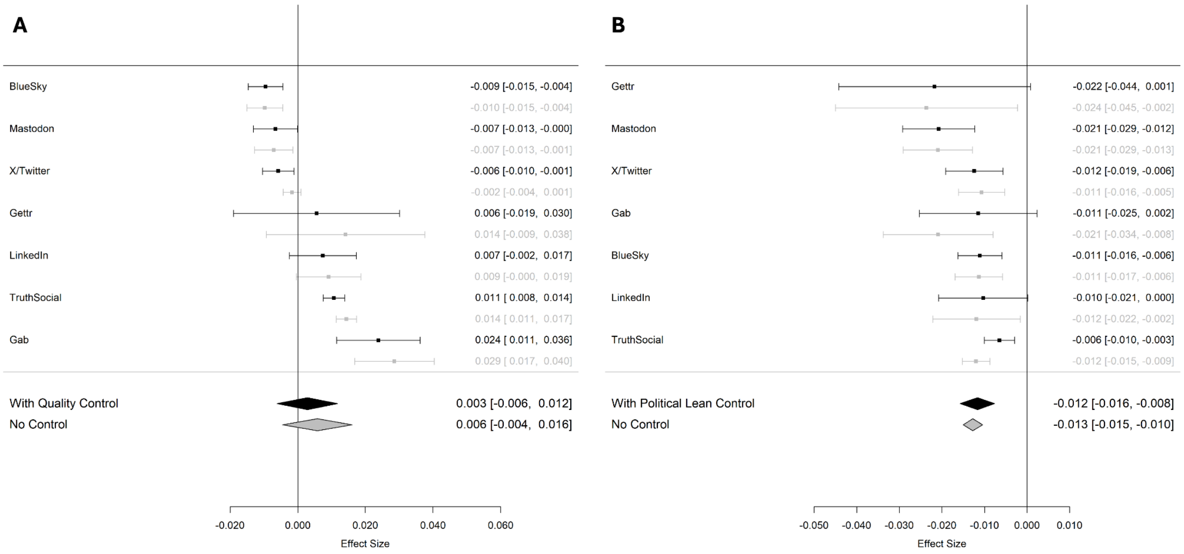

Figure S14. Robustness of results predicting likes only. *Linear regression predicting log-transformed number of likes of (A) news domain political lean, and (B) news domain quality, with or without controlling for news domain quality/political lean; all models include user fixed effects.*

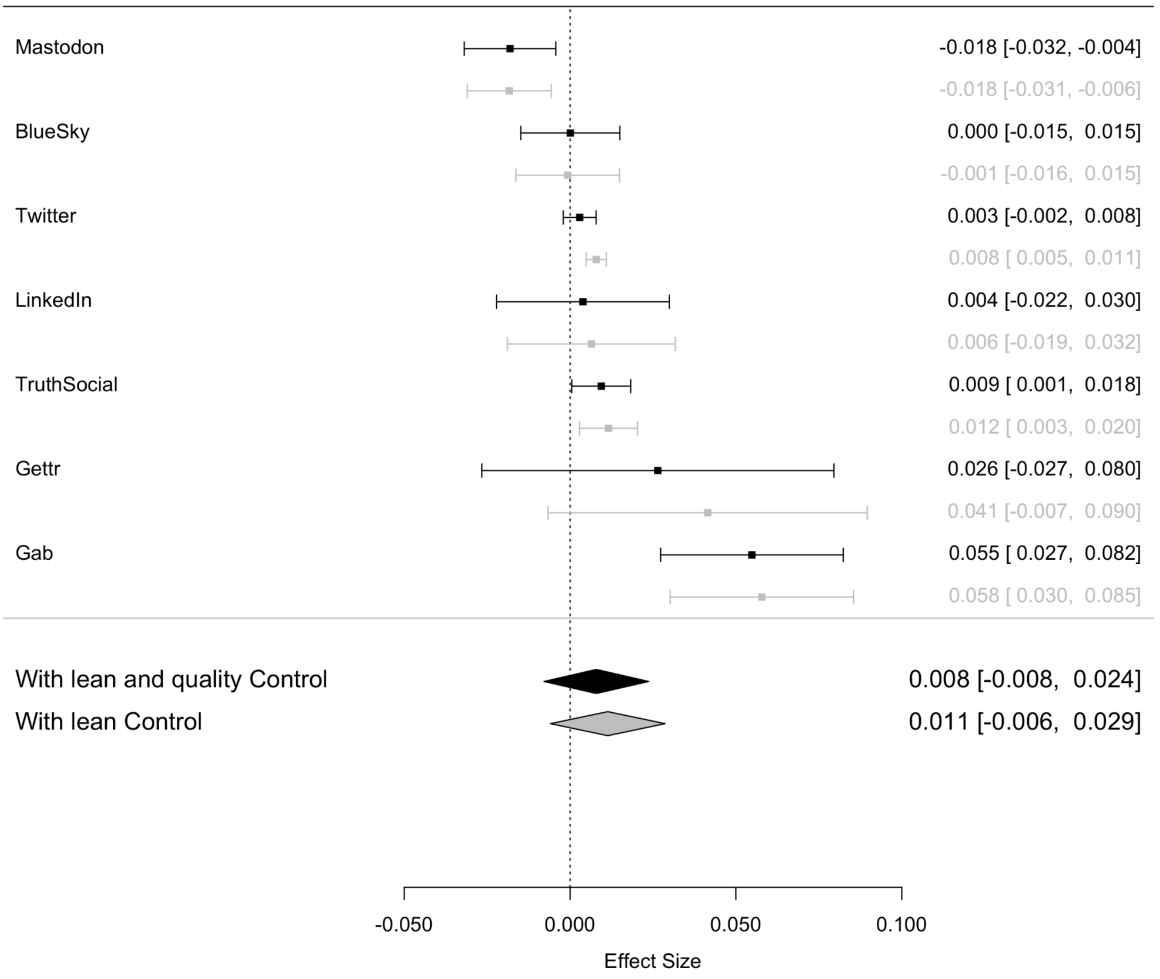

Figure S15. Association between engagements and partisan news across platforms. *Linear regression predicting log-transformed engagement using dummy for partisan vs neutral news domain (defined as partisan =abs(political lean) > 0.5), with controlling for news domain political lean or controlling for news domain political lean and quality; all models include user fixed effects.*

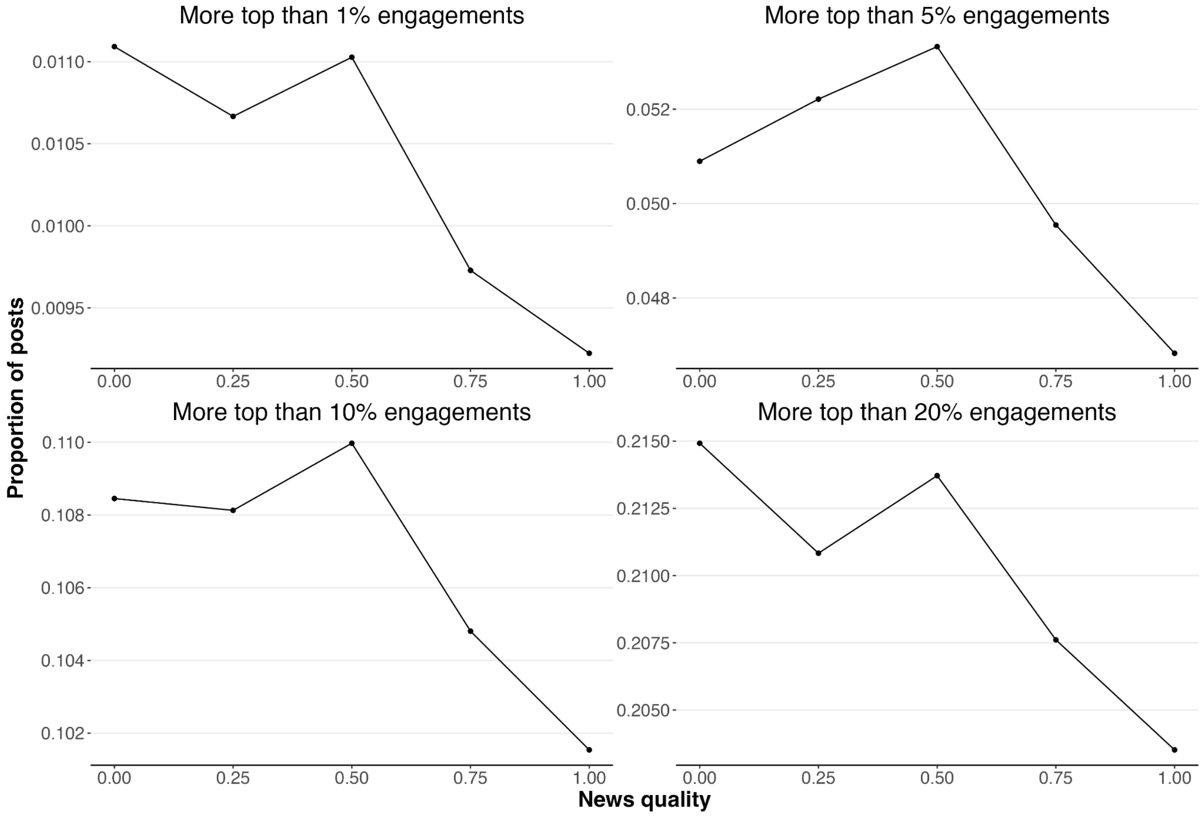

Figure S16. Proportion of posts that are among the X% highest engagement posts on each platform by news quality (binned by rounding quality to the nearest 0.25).

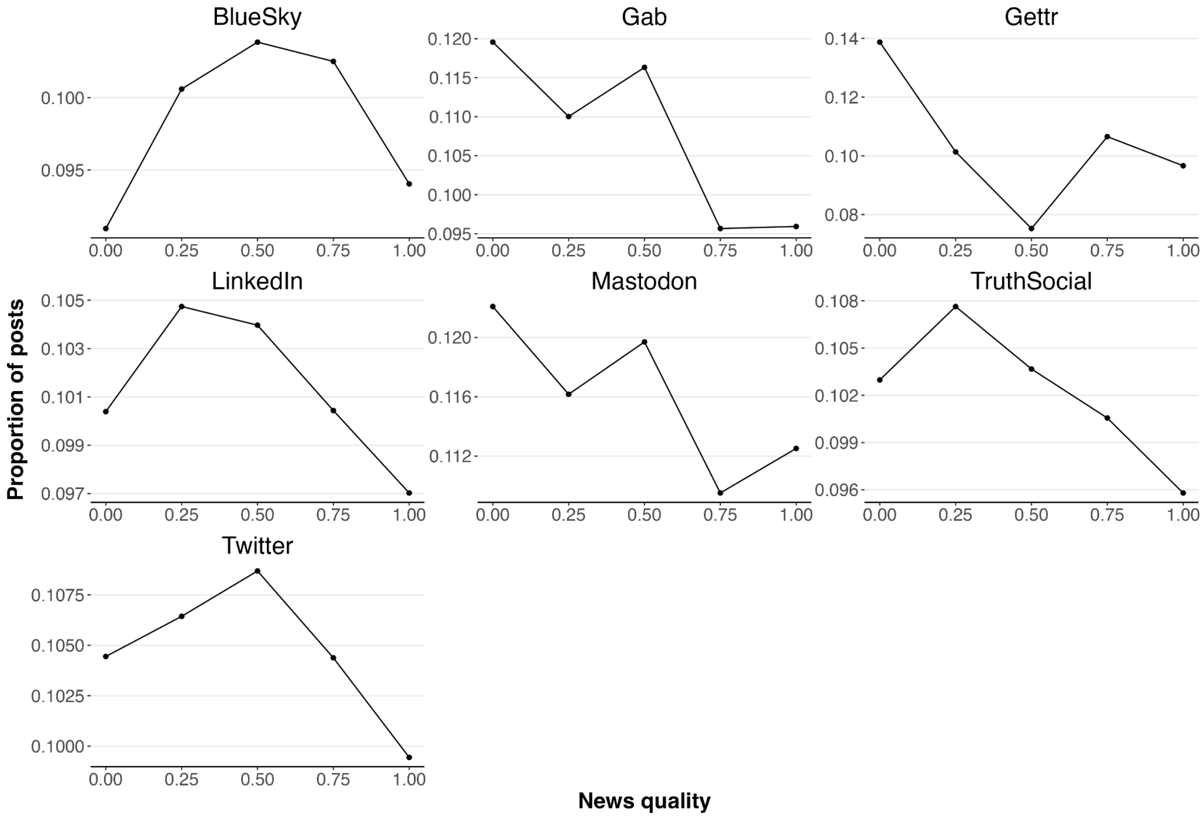

Figure S17. Proportion of posts that are among the 10% highest engagement posts on each platform by news quality (binned by rounding quality to the nearest 0.25).

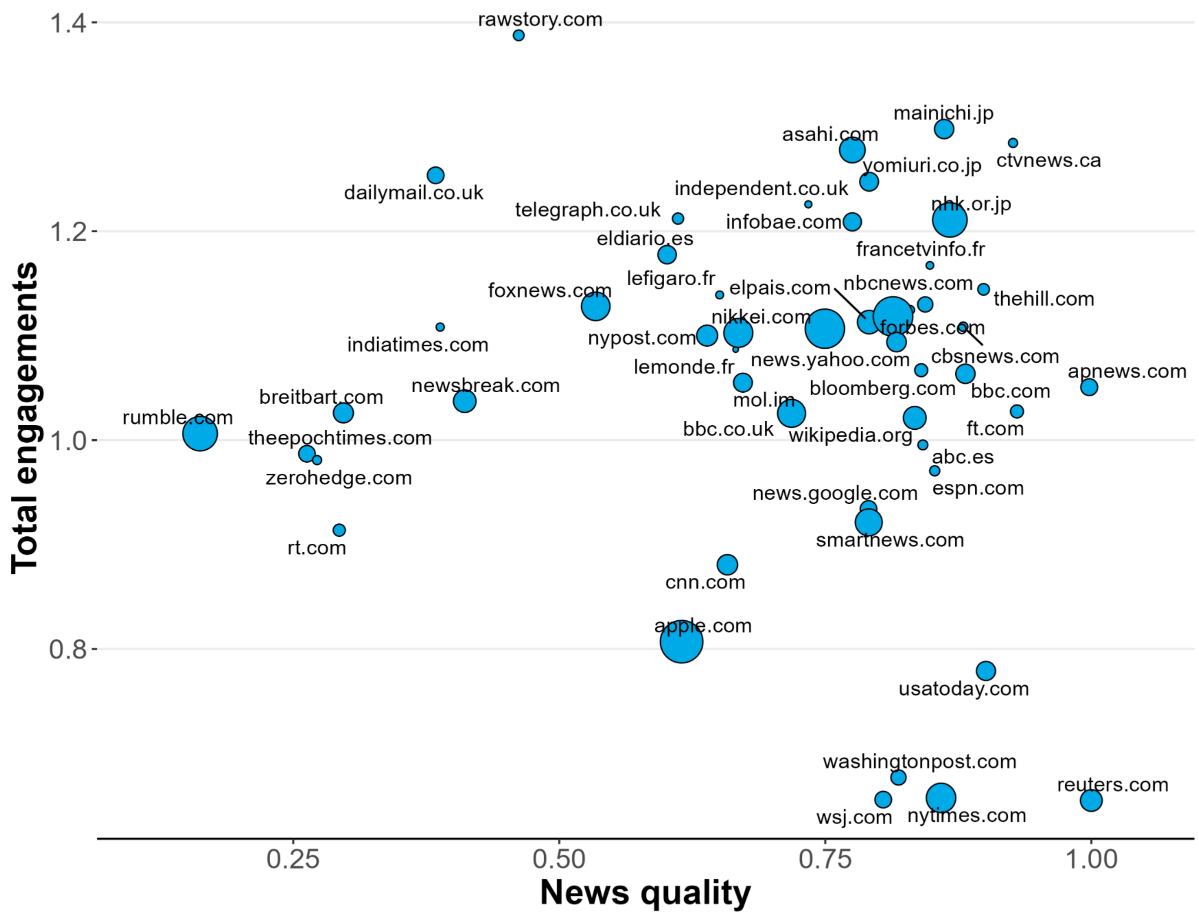

Figure S18. Average total engagement per post of top 50 most-frequently shared news domains versus quality of content.

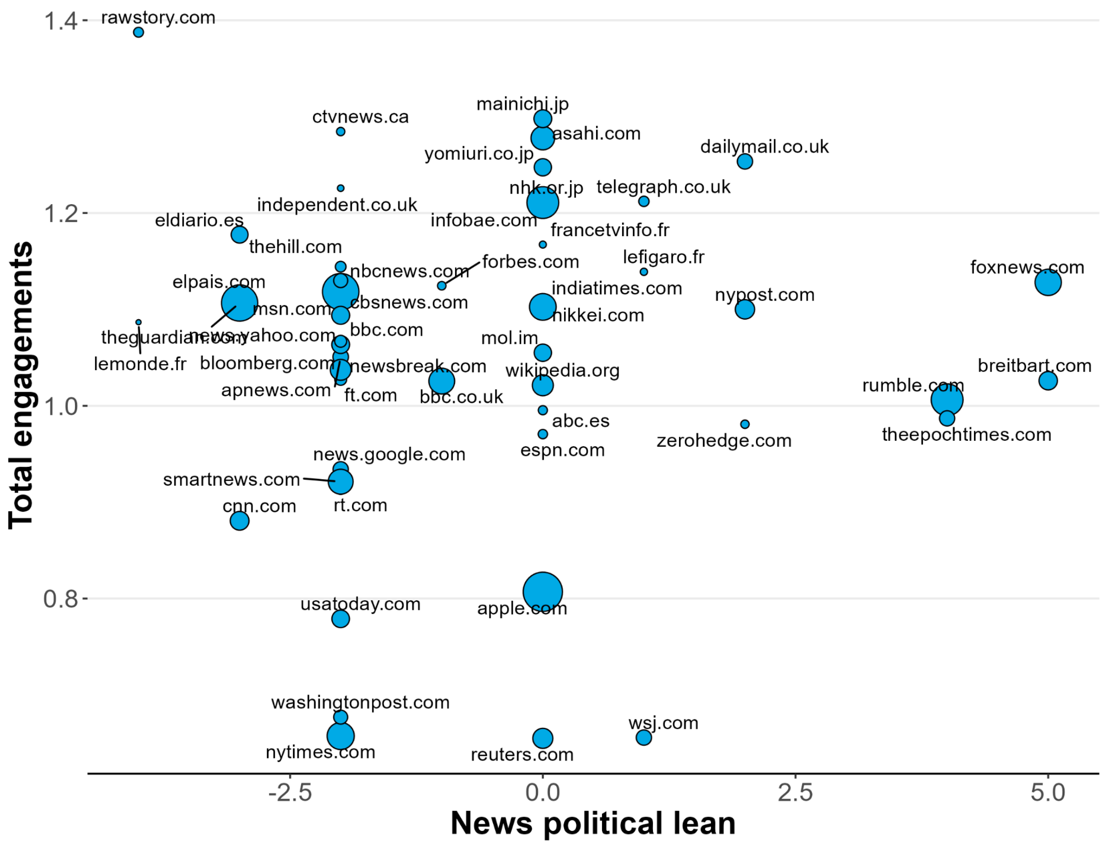

Figure S19. Average total engagement per post of top 50 most-frequently shared news domains versus news domain political lean.

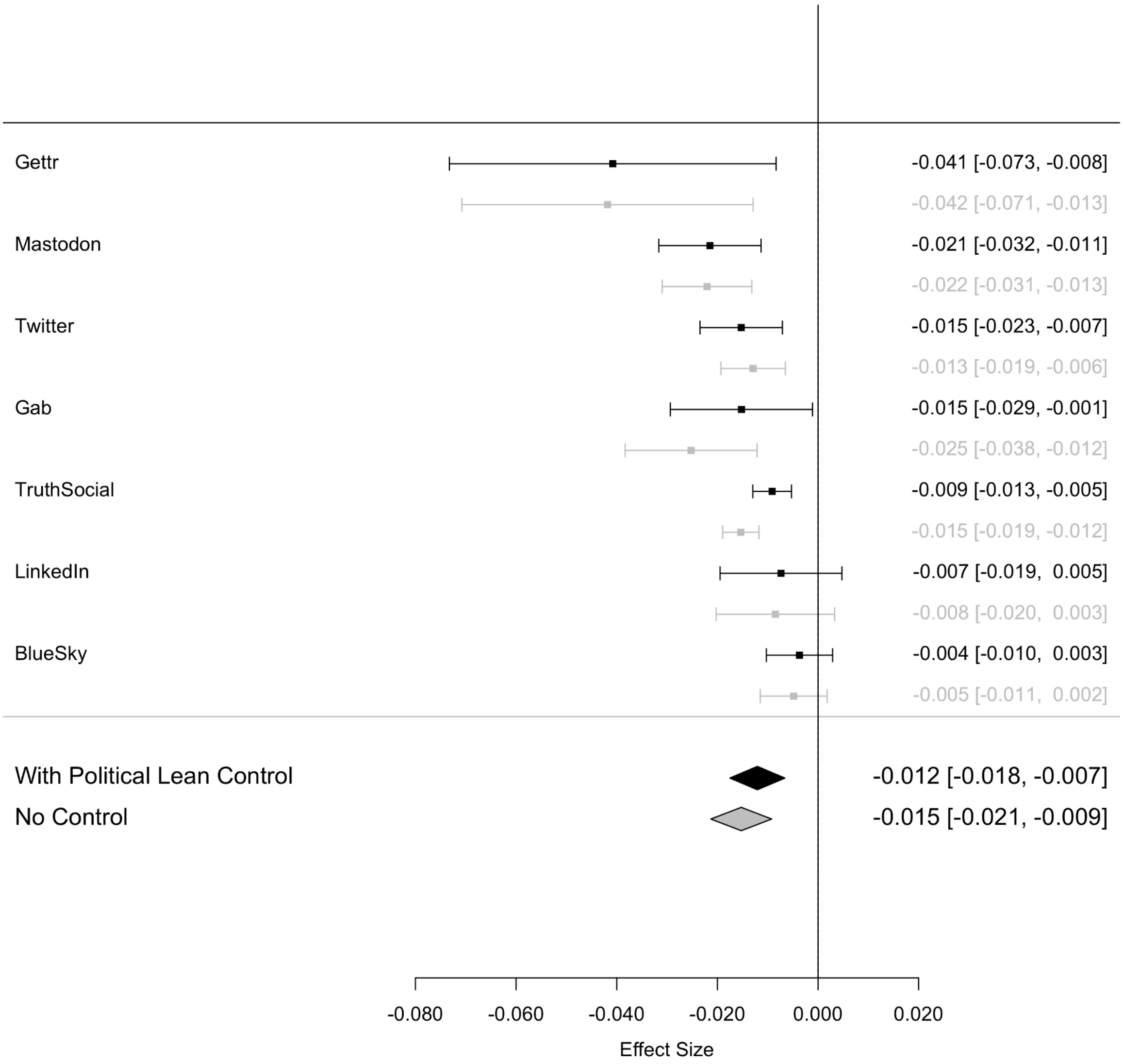

Figure S20. Association between engagement and quality of news across platforms removing non-English posts. *Linear regression predicting log-transformed engagement of news domain, with or without controlling for news domain political lean; all models include user fixed effects.*

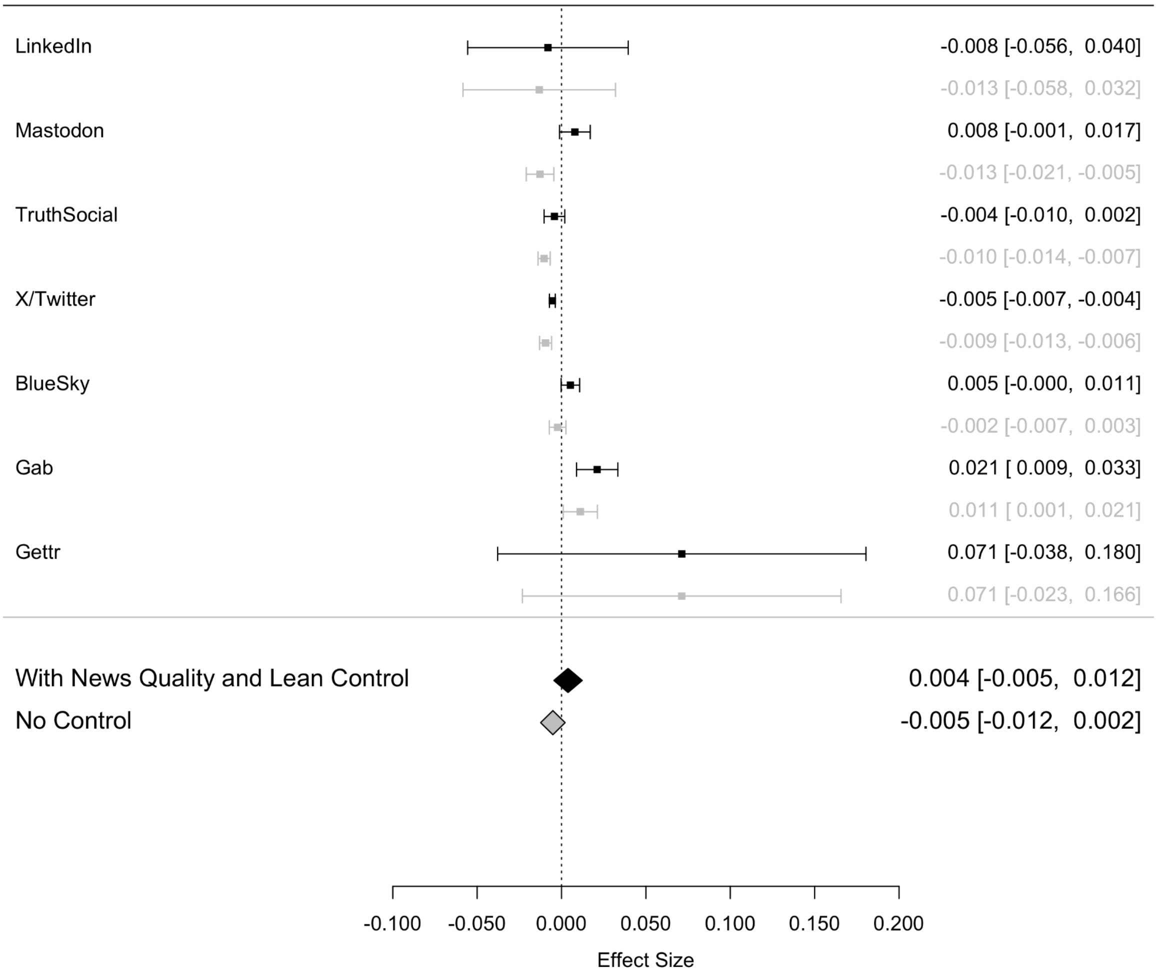

Figure S21. Association between engagement and the distance between the political lean of a news domain and the average political lean of news shared on a platform. *We defined distance as the difference between the political lean of the news domain and the average political lean of the platform with its direction being the same as the average political lean of the platform. The results, although not conclusive, suggest that extreme content receives more engagement on echo-chambered platforms, whereas extreme content receives less engagement on town-square platforms (X and LinkedIn)*

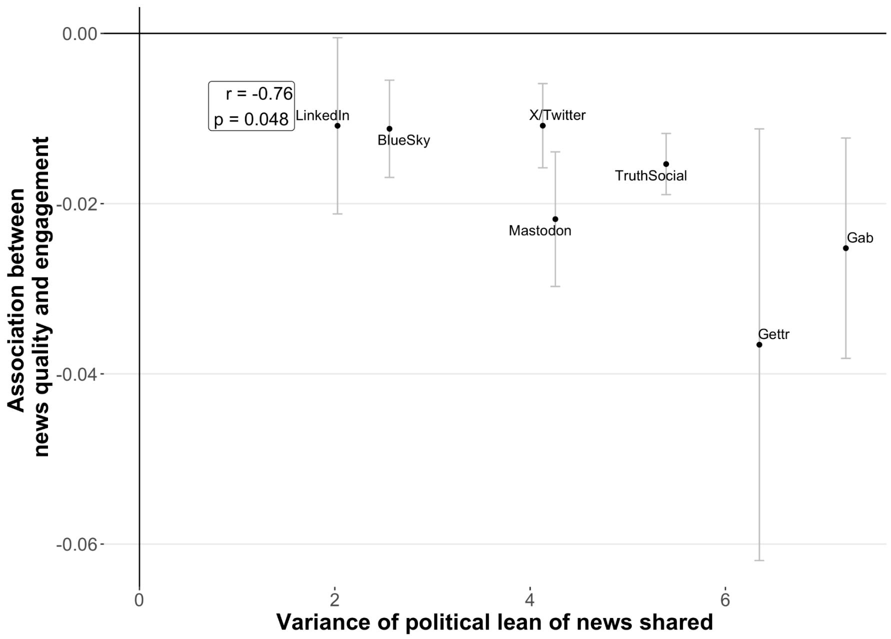

Figure S22. Association between news quality and engagement versus variance of political lean of news shared.

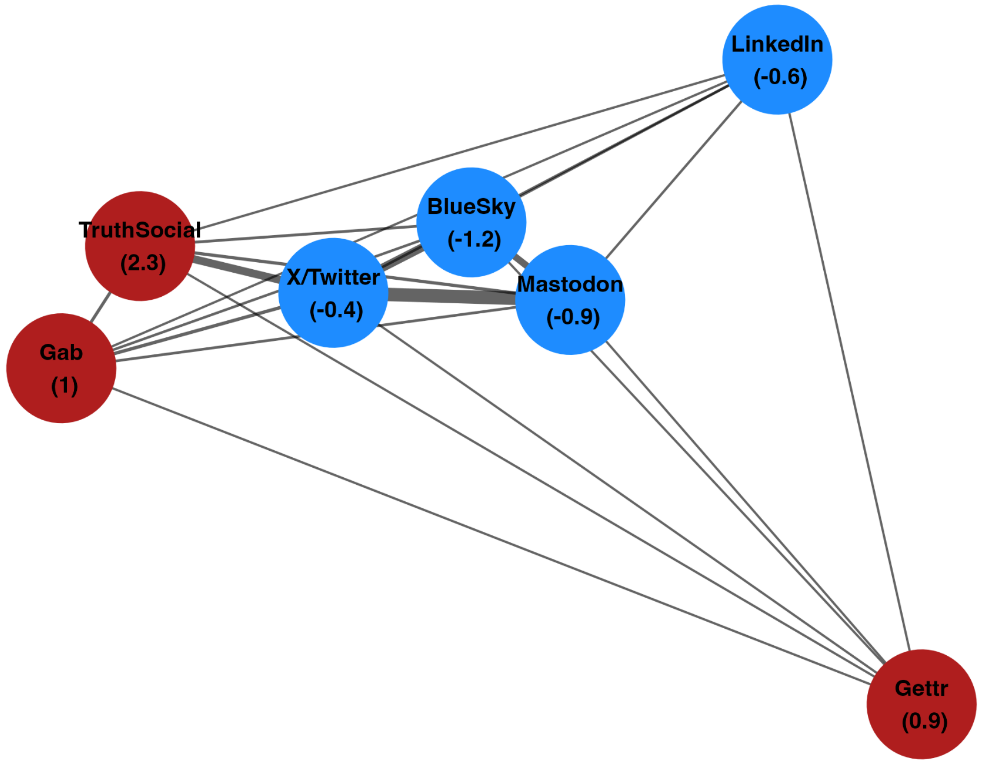

Figure S23. Co-share network of news domains across platforms. *Edge weights are calculated as the sum of minimum shared counts for each domain to reflect co-appearance intensity. Nodes are positioned using a force-directed layout, with nodes connected by larger edges placed closer together. Node color indicates the average political leaning of news domains shared (red = Republican, blue = Democrat), and the average political value is annotated on each node.*

Table S1. Top 5 frequently shared domains in each quality category in our sample for each platform. *For each level of quality top 5 mostly shared domains are shown in order.*

News quality rounded by .25 bins				
**0**	**.25**	**.5**	**.75**	**1**
**BlueSky**				
bitchute.com	postillon.com	sueddeutsche.de	tagesschau.de	nature.com
thegatewaypundit.com	mondoweiss.net	n-tv.de	nbcnews.com	npr.org
activistpost.com	rumble.com	ndr.de	zeit.de	reuters.com
infowars.com	express.co.uk	deutschlandfunk.de	spiegel.de	zdf.de
scenarieconomici.it	wonkette.com	rollingstone.com	taz.de	theverge.com
**Gab**				
prepareforchange.net	tanker-enemy.com	peacock-panache.com	herald-review.com	herald-dispatch.com
rense.com	aurora-news.us	theintercept.com	time.com	tri-cityherald.com
thegatewaypundit.com	archaeology-world.com	humanevents.com	news.sky.com	fortune.com
disclose.tv	tg-quotidiano.net	people.com	reason.com	arstechnica.com
infowars.com	usawatchdog.com	amac.us	forward.com	theconversation.com
**GETTR**				
geoengineeringwatch.org	express.co.uk	americasvoice.news	bloomberg.com	rescue.org
nationalfile.com	redstate.com	freebeacon.com	weforum.org	bbc.com
newstarget.com	childrenshealthdefense.org	dailycaller.com	ncbi.nlm.nih.gov	congress.gov
prepareforchange.net	judicialwatch.org	bizpacreview.com	welt.de	usatoday.com
educate-yourself.org	zerohedge.com	amgreatness.com	cnn.com	axios.com
**LinkedIn**				
creation.com	express.co.uk	public.fr	bing.com	ft.com
beforeitsnews.com	mindbodygreen.com	apple.com	health.com	ieee.org
gaia.com	rt.com	newsbreak.com	cancer.org	nature.com
infowars.com	rumble.com	si.com	statista.com	azcentral.com
newstarget.com	newsmax.com	history.com	news.com.au	cleveland.com
**Mastodon**				
bitchute.com	rumble.com	ndr.de	theguardian.com	bbc.com
thegatewaypundit.com	brighteon.com	deutschlandfunk.de	nytimes.com	arstechnica.com
naturalnews.com	zerohedge.com	sueddeutsche.de	tagesschau.de	theverge.com
infowars.com	theepochtimes.com	rollingstone.com	bbc.co.uk	apnews.com
activistpost.com	mondoweiss.net	change.org	washingtonpost.com	reuters.com
**TruthSocial**				
nationalfile.com	thenationalpulse.com	thepostmillennial.com	abcnews.go.com	thehill.com

beforeitsnews.com	revolver.news	dailycaller.com	news.yahoo.com	apnews.com
naturalnews.com	thefederalist.com	babylonbee.com	conservativebrief.com	fortune.com
prepareforchange.net	redstate.com	trendingpoliticsnews.com	time.com	ballotpedia.org
thegatewaypundit.com	townhall.com	lawenforcementtoday.com	americanmilitarynews.com	patch.com
**X/Twitter**				
bitchute.com	rumble.com	apple.com	theguardian.com	bbc.com
infowars.com	theepochtimes.com	foxnews.com	nhk.or.jp	reuters.com
naturalnews.com	breitbart.com	dailymail.co.uk	nytimes.com	apnews.com
disclose.tv	zerohedge.com	telegraph.co.uk	wikipedia.org	thehill.com
beforeitsnews.com	rt.com	finance.yahoo.com	bbc.co.uk	cbsnews.com

Table S2. Controlling for paywalled news domains. *Predicting log-transformed number of engagements plus one across all platforms using news domain quality rating, political lean, dummy variable representing if the domain is paywall and its interaction with news quality. Results are generated using a linear regression model with user fixed effects.*

News quality	-0.011***
	(0.003)
Paywall	-0.008***
	(0.001)
News quality*Paywall	-0.008***
	(0.002)
Political lean	-0.005**
	(0.002)

Table S3. Results for Telegram. *Predicting log-transformed number likes plus one on Telegram using news domain quality rating and political lean. Results are generated using a linear regression model with user fixed effects.*

	(1)	(2)
News quality	-0.021***	-0.024***
	(0.005)	(0.010)
Political lean		0.006
		(0.005)

Table S4. List of non-news domains excluded from the list of rated domains.

Domains
"youtube.com", "youtu.be", "youtube.be", "t.me", "yt.be", "facebook.com", "google.com", "yahoo.com", "blogspot.com", "twitter.com", "wordpress.com", "medium.com", "instagram.com", "substack.com", "spotify.com", "spoti.fi", "walmrt.us", "walmart.com", "tiktok.com", "fb.watch", "paypal.com", "gofundme.com", "linkedin.com", "patreon.com", "twitch.tv", "reddit.com", "blog.jp", "zoom.us", "amazon.com", "amazon.ca", "amazon.co.jp", "amazon.co.uk", "dailymotion.com", "telegram.org", "discord.gg", "apple.news", "yahoo.com", "sankei.com", "nordot.app", "anonymous-post.mobi", "odysee.com", "gettr.com"

1. M. Mosleh, D. G. Rand, Measuring exposure to misinformation from political elites on Twitter. *Nature Communications* **13**, 7144 (2022).
2. P. Barberá, J. T. Jost, J. Nagler, J. A. Tucker, R. Bonneau, Tweeting from left to right: Is online political communication more than an echo chamber? *Psychological science* **26**, 1531–1542 (2015).
3. N. Grinberg, K. Joseph, L. Friedland, B. Swire-Thompson, D. Lazer, Fake news on Twitter during the 2016 US presidential election. *Science* **363**, 374–378 (2019).
4. G. Eady, R. Bonneau, J. A. Tucker, J. Nagler, News Sharing on Social Media: Mapping the Ideology of News Media, Politicians, and the Mass Public. (2020).
